# ACP-TX-I and ACP-TX-II, Two Novel Phospholipases A_2_ Isolated from Trans-Pecos Copperhead *Agkistrodon contortrix pictigaster* Venom: Biochemical and Functional Characterization

**DOI:** 10.3390/toxins11110661

**Published:** 2019-11-14

**Authors:** Salomón Huancahuire-Vega, Luciana M. Hollanda, Mauricio Gomes-Heleno, Edda E. Newball-Noriega, Sergio Marangoni

**Affiliations:** 1 Departamento de Ciencias Básicas, Facultad de Ciencias de la Salud, Escuela de Medicina Humana, Universidad Peruana Unión (UPeU), Lima 15, Peru; eddanewball@upeu.edu.pe; 2 Instituto de Tecnologia e Pesquisa, Universidade Tiradentes (UNIT), Aracaju 49032-490, SE, Brazil; luciana.hollanda@gmail.com; 3 Departamento de Bioquímica, Instituto de Biologia, Universidade Estadual de Campinas (UNICAMP), Campinas 13083-970, SP, Brazil; maoheleno@yahoo.com.br (M.G.-H.); marango@unicamp.br (S.M.)

**Keywords:** snake venom, *Agkistrodon contortrix pictigaster*, D49 PLA_2_, homologous K49 PLA_2_, myotoxin, edema-forming activity and cytotoxicity

## Abstract

This work reports the purification and biochemical and functional characterization of ACP-TX-I and ACP-TX-II, two phospholipases A_2_ (PLA_2_) from *Agkistrodon contortrix pictigaster* venom. Both PLA_2_s were highly purified by a single chromatographic step on a C_18_ reverse phase HPLC column. Various peptide sequences from these two toxins showed similarity to those of other PLA_2_ toxins from viperid snake venoms. ACP-TX-I belongs to the catalytically inactive K49 PLA_2_ class, while ACP-TX-II is a D49 PLA_2_, and is enzymatically active. ACP-TX-I PLA_2_ is monomeric, which results in markedly diminished myotoxic and inflammatory activities when compared with dimeric K49 PLA_2_s, confirming the hypothesis that dimeric structure contributes heavily to the profound myotoxicity of the most active viperid K49 PLA_2_s. ACP-TX-II exhibits the main pharmacological actions reported for this protein family, including in vivo local myotoxicity, edema-forming activity, and in vitro cytotoxicity. ACP-TX-I PLA_2_ is cytotoxic to A549 lung carcinoma cells, indicating that cytotoxicity to these tumor cells does not require enzymatic activity.

## 1. Introduction

Phospholipases A_2_ (PLA_2_), which hydrolyze 2-acyl ester bonds of 3-sn-phospholipids, releasing lysophospholipids and fatty acids, are widespread in snake venoms, facilitating the immobilization and digestion of prey [1,2]. Venom PLA_2_s belong to the secretory PLA_2_ (sPLA_2_) family (groups IA, elapids and IIA, viperids) [3,4]. PLA_2_s are small proteins (13–15 kDa) with 115–122 residues, and seven conserved disulfide bonds [5,6]. They are subdivided into two main subgroups: (1) D49 PLA_2_s, which have an aspartate residue at position 49, and typically have high catalytic activity [7,8], and (2) K49 PLA_2_s, with a lysine residue at position 49. These have little or no catalytic activity, but still induce various biological effects [9,10]. Both types of proteins show significant similarity in their three-dimensional structures, although they exhibit different pharmacological actions, such as myotoxicity, neurotoxicity, anticoagulant activity, platelet aggregation inhibition/activation, hemolysis, edematogenicity, hypotension, bactericidal action, proinflammatory cytotoxicity, and antitumor activity [2,11].

A great number of sequences and crystal structures of viperid PLA_2_s have been determined [11,12,13]. Despite their overarching similarities, sequence differences result in diverse biological functions [14]. Accordingly, characterization of individual toxins is important to better understand the pathophysiology of envenomation and to potentially improve therapeutic procedures [15].

*Agkistrodon* is a genus of pit vipers ranging from the southern United States to northern Costa Rica [16]. Currently, this genus comprises four species: *A. contortrix* (copperheads), *A. piscivorus* (cottonmouth), *A. bilineatus* (cantils), and *A. taylori* (Taylor’s cantils) [17,18]. Copperheads comprise several subspecies: *A. c. contortrix* (southern copperhead), *A. c. laticinctus* (broad-banded copperhead), *A. c. mokasen* (northern copperhead), *A. c. phaeogaster* (Osage copperhead), and *A. c. pictigaster* (Trans-Pecos copperhead). Subspecific taxonomy is based largely on gross morphology, color pattern, and scale counts [18]. Cottonmouths and copperheads are among the most common venomous snakes in the southeastern United States. Cottonmouths frequent streams, rivers, ponds, marshes, and swamps, whereas copperheads are found in deciduous hardwood forests with moist leaf litter, large logs, scattered rocks, and high levels of vegetative cover. These snakes account for ~30% of the non-lethal human envenomations in this region [19,20].

The Trans-pecos copperhead (*A. c. pictagaster*) is found in western Texas, northern Chihuahua, and Coahuila (Mexico) [16]. Juveniles usually prey on invertebrates (spiders, millipedes, and insects), frogs, and small lizards, whereas adults primarily prey on vertebrates, including amphibians (salamanders and anurans), reptiles (lizards and snakes), birds, and small mammals (rodents) [16].

Studies on the biochemical composition and toxic activities of copperhead venoms, including D49 PLA_2_ and homologous K49 PLA_2_, have been almost exclusively restricted to *A. c. contortrix* and *A. c. latincictus*, [5,21,22,23]. In contrast, little information is available on *A. c. pictigaster*. Partial characterizations of two disintegrins from this venom have been described by Lucena et al. [24]. A comparison of venom proteome variation in the genus *Agkistrodon* found that *A. c. pictigaster* venom contains ten protein families, dominated by PLA_2_s (38.2%) and metalloproteinases (30.2%). The venom showed proteolytic, hemorrhagic, and myotoxic activities [25].

This work is the first report of two basic PLA_2_s isolated from *A. c. pictigaster* venom, with their identification and structural characterization found by biochemical and enzymatic experiments. Furthermore, we describe their biological activities and cytotoxic properties upon an A549 tumor cell line. The results of this study illuminate structure-function relationships of ACP-TX-I and ACP-TX-II PLA_2_, and improve our understanding of the chemistry of this venom.

## 2. Results

### 2.1. Purification and Biochemical Characterization of ACP-TX-I and ACP-TX-II

**Figure 1:**
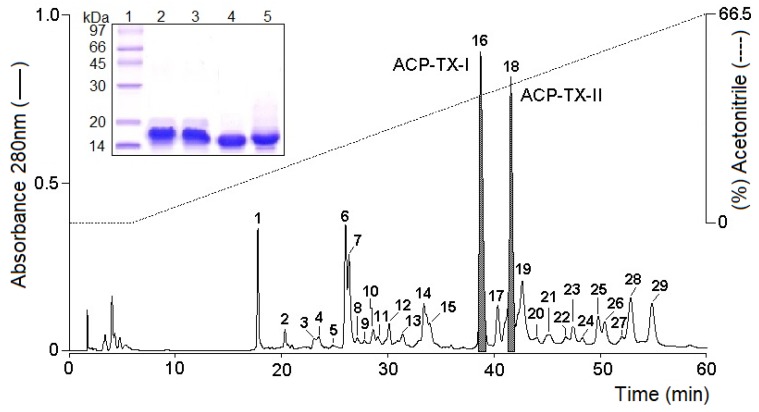
When *A. c. pictigaster* venom was fractionated on a C_18_ µ-Bondapak column, two phospholipases A_2_ dominated the elution profile. Fraction 18 (ACP-TX-II) possessed PLA_2_ activity, while fraction 16 (ACP-TX-I) showed cytotoxic activity, despite a lack of enzymatic activity. Insert: Electrophoretic profile in Tricine SDS-PAGE. (1) Molecular mass markers; (2) ACP-TX-I not reduced; (3) ACP-TX-I reduced with DTT (1M); (4) ACP-TX-II not reduced; and (5) ACP-TX-II reduced with DTT (1M).

Chromatographic separation of *A. c. pictigaster* venom by reversed phase high-performance liquid chromatography (RP-HPLC) on a C_18_ column resulted in 29 fractions, with two prominent peaks (16 and 18) eluting in more than 30% acetonitrile (Figure 1). These peaks, representing about 30% of total venom protein, were collected and screened for PLA_2_ activity. Fractions 16 and 18 were named ACP-TX-I and ACP-TX-II, respectively. Both ACP-TX-I and ACP-TX-II exhibited high purity when re-chromatographed using the same chromatography system, each showing only one peak (Figure S1). These peaks were also analyzed by SDS-PAGE, which manifested a single electrophoretic band with an *Mr* of approximately 14 kDa under reducing and non-reducing conditions (Figure 1 insert).

**Figure 2:**
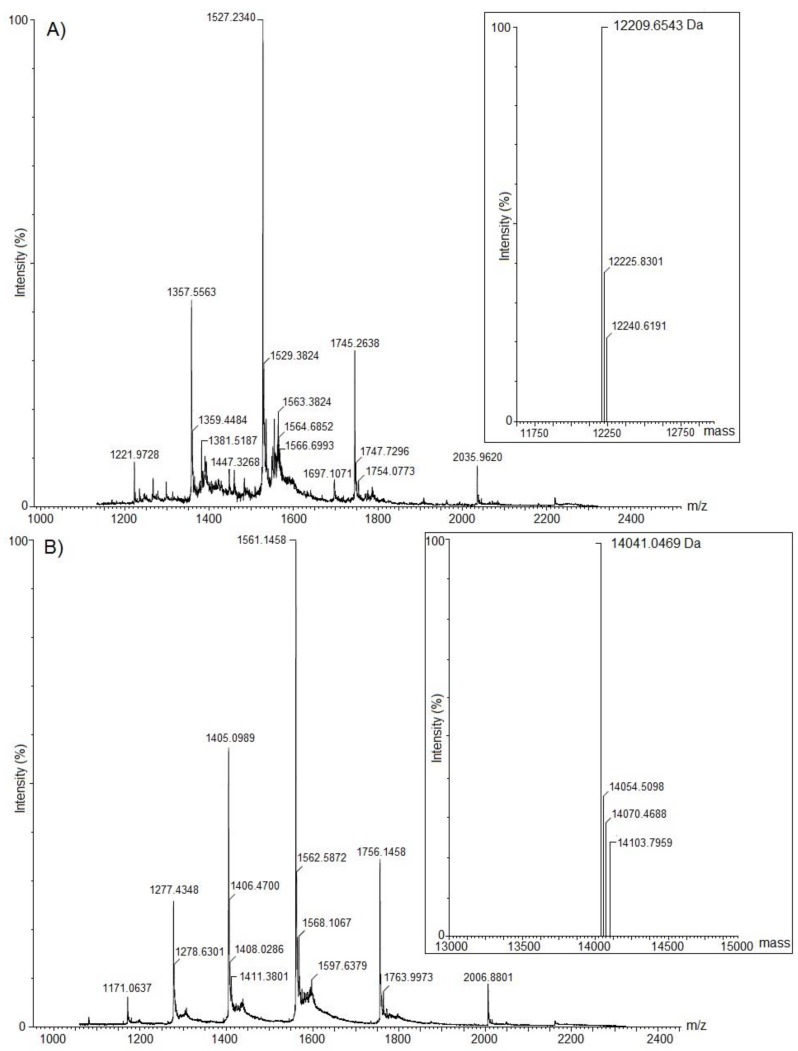
Molecular mass determination of ACP-TX-I (**A**) and ACP-TX-II (**B**) by nanoelectrospray tandem mass spectrometry using a Quadrupole Time-of-flight (Q-Tof) Ultima API mass spectrometer (MicroMass/Waters) with an output mass range of 6000–20,000 Da at a “resolution” of 0.1 Da/channel. Raw and deconvoluted electrospray mass spectra are shown (inserts).

ESI-MS analysis demonstrated that the proteins were homogeneous, with molecular masses of 12,209.7 and 14,041.1 Da for ACP-TX-I and ACP-TX-II, respectively (Figure 2A,B).

### 2.2. Determination of the Amino Acid Sequences of ACP-TX-I and ACP-TX-II

In order to identify the purified proteins, they were digested with trypsin, and tryptic peptides were detected and characterized by mass spectrometry. Amino acid sequences of several tryptic peptides were obtained (Table 1). ACP-TX-I and ACP-TX-II shared 7 and 6 peptides with other viperid PLA_2_s, respectively.

**Figure 3:**
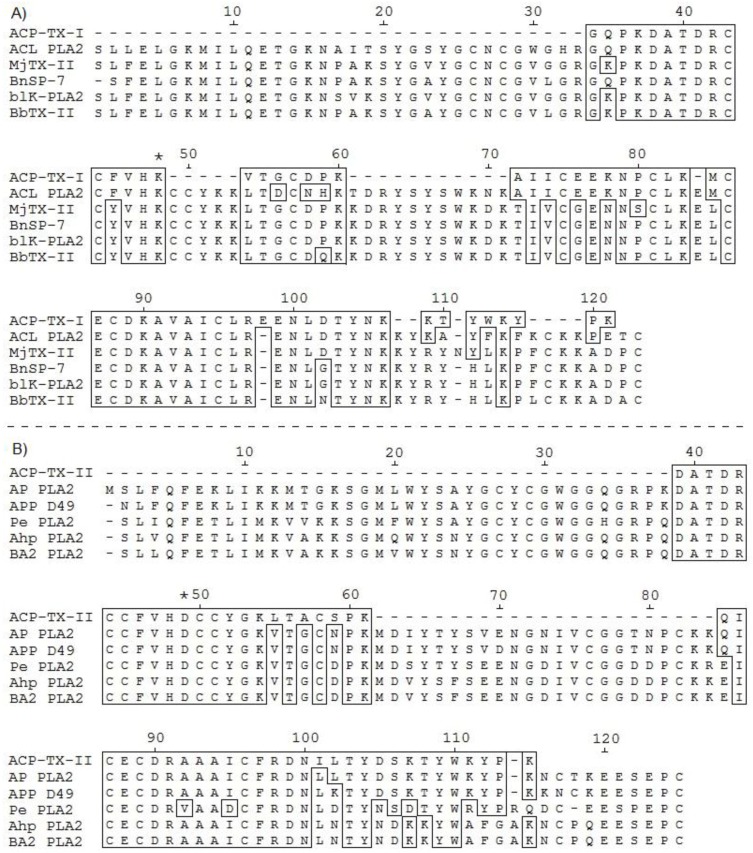
ACP-TX-I (**A**) and ACP-TX-II (**B**) show significant similarity to K49 and D49 PLA_2_s, respectively (Edit Seq version 5.01© Program, DNASTAR Inc., Madison, WI, USA, 2001). ACL from *A. c. laticinctus* [26]; MjTX-II from *B. moojeni* [27]; BnSP-7 from *B. neuwiedi pauloensis* [28]; blK PLA_2_ from *B. leucurus* [29]; BbTX-II from *B. brazili* [6]; AP PLA_2_ from *A. piscivorus* [30]; APP PLA_2_ from *A. p. piscivorus* [31]; Pe PLA_2_ from *Protobothrops elegans* [32]; Ahp and BA2 PLA_2_ from *A. halys pallas* [33,34]. Hyphens indicate gaps generated by the alignment software.

Assembly of partial protein sequences by similarity, using BLAST and multiple alignment, demonstrated that ACP-TX-I is a K49 PLA_2_ (Figure 3A), and is quite similar to well-characterized members of this family such as ACL PLA_2_ from *A. c. laticinctus*, MjTX-II from *Bothrops moojeni*, BnSP-7 from *B. neuwiedi pauloensis*, etc. Sequenced peptides accounted for 64 amino acids, assuming that the number of residues is identical to that of homologous viperid toxins. This represented an estimated 53% of the protein (Figure 3A). ACP-TX-II shares conserved domain sequences common to catalytically active D49 PLA_2_s. Peptide 1, having the sequence DATDRCCFVHDCCYGQ/K, contains an Asp (aspartic acid) residue that corresponds to position 49 in the complete amino acid sequence (Figure 3B).

### 2.3. Activity Measurements of ACP-TX-II

**Figure 4:**
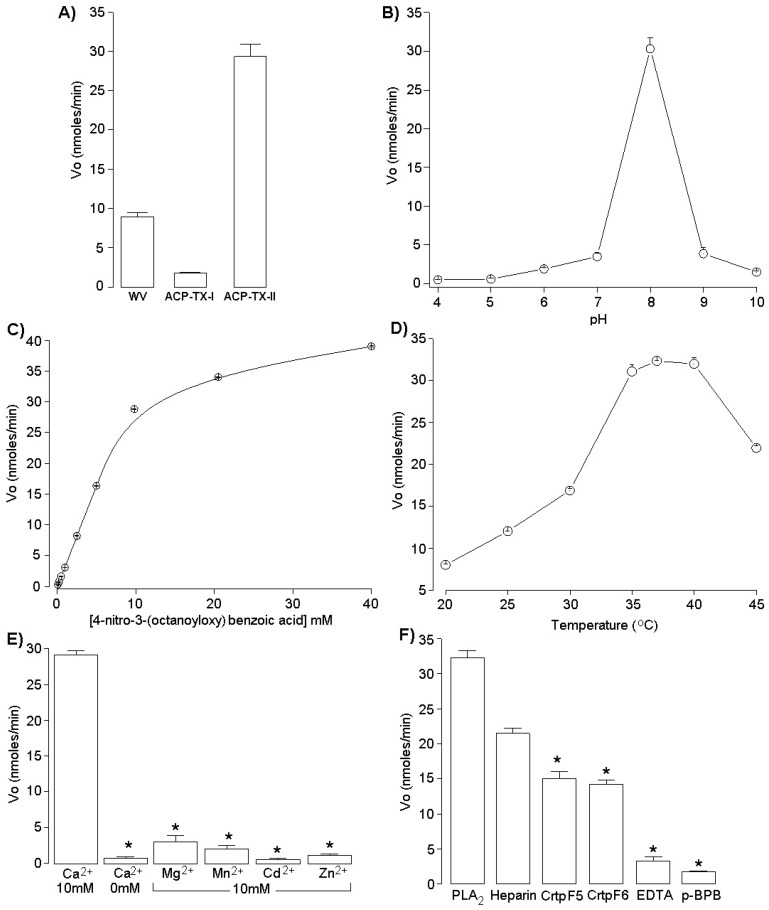
(**A**) PLA_2_ activity of *A. c. pictigaster* venom, ACP-TX-I and ACP-TX-II PLA_2_; (**B**) Effect of pH on the PLA_2_ activity of ACP-TX-II; (**C**) Effect of substrate concentration on the PLA_2_ activity of ACP-TX-II; (**D**) Effect of temperature on the PLA_2_ activity of ACP-TX-II; (**E**) Influence of ions (10 mM each) on PLA_2_ activity of ACP-TX-II; (**F**) Effect of heparin, EDTA crotapotins (F5 and F6) and chemical modification with BPB on PLA_2_ activity of ACP-TX-II. The results are the mean ± SEM of three determinations (* *p* < 0.05).

ACP-TX-I did not show PLA_2_ activity, but possessed a mass of ~14 kDa. ACP-TX-II displayed specific PLA_2_ activity of 29.31 ± 1.62 nmol/min (Figure 4A). The pH optimum was 8.0 (Figure 4B) and this protein was stable at temperatures from 35 to 40 °C (Figure 4D). At low concentrations, ACP-TX-II showed a sigmoidal relationship with temperature (Figure 4C) and a strict dependence on calcium ions (10 mM) for full activity. Substitution of Ca^2+^ with Mg^2+^, Mn^2+^, Cd^2+^, or Zn^2+^ (10 mM) significantly reduced enzyme activity (Figure 4E). Enzymatic activity of ACP-TX-II was abolished by EDTA and treatment with *p*-BPB. Incubation with crotapotins, F5 and F6, from *C. d. collilineatus* venom also diminished activity, while heparin did not significantly inhibit catalysis (Figure 4F).

### 2.4. Pharmacological Activities of ACP-TX-I and ACP-TX-II

**Figure 5:**
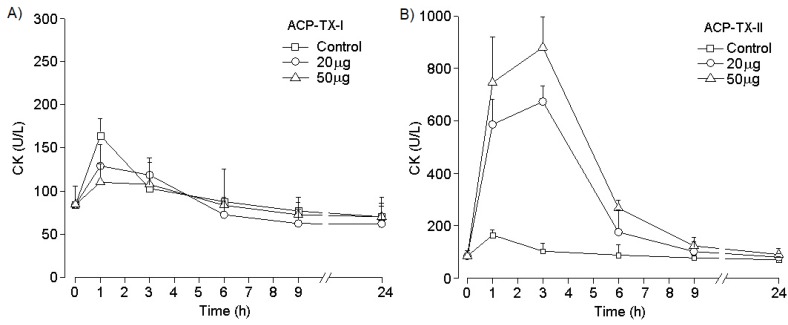
Myotoxic activity of ACP-TX-I PLA_2_ (**A**) and ACP-TX-II PLA_2_ (**B**) in mice. Time-course of the increments in plasma CK activity after an intramuscular injection of 20 and 50 µg of toxins compared to the injection of vehicle alone (PBS). Points represent means ± SD of four mice per group.

In vivo, ACP-TX-II PLA_2_ (20 and 50 µg), injected intramuscularly (IM), induced local myonecrosis, and time-course analysis showed a maximum increase in plasma CK 3 h after injection, returning to normal by 24 h (Figure 5B). In contrast, ACP-TX-I PLA_2_ showed no local myotoxic effect, even at high concentrations (Figure 5A). ACP-TX-I and ACP-TX-II PLA_2_ did not show systemic myotoxicity after intravenous (IV) injection (Figure S2).

**Figure 6:**
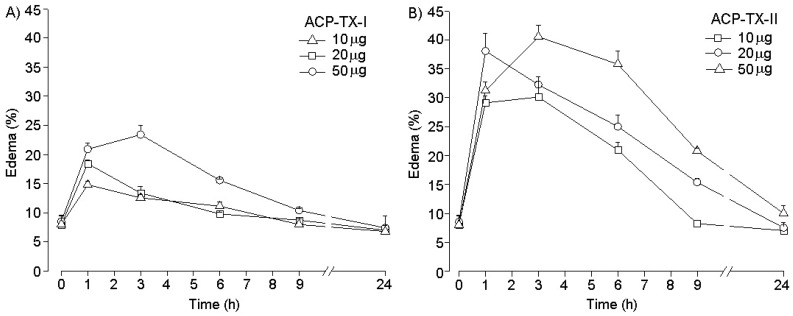
Edema-forming activity of ACP-TX-I PLA_2_ (**A**) and ACP-TX-II PLA_2_ (**B**) in mice. Induction of edema by toxins (10, 20 and 50 µg), injected SC in the footpads of mice. At various time intervals, the increase in footpad volume, compared to controls, was expressed as percent edema. Each point represents the mean ± SD of four animals per group.

ACP-TX-I PLA_2_ had little edematogenic effect, since a 50-µg injection was necessary in order to observe 23.4% edema after 3 h (Figure 6A). In contrast, ACP-TX-II PLA_2_ presented marked paw edema, with maximal activity (40.54%) 3 h after a 50-μg injection. Edema returned to normal levels after 24 h, showing dose-dependent activity.

**Figure 7:**
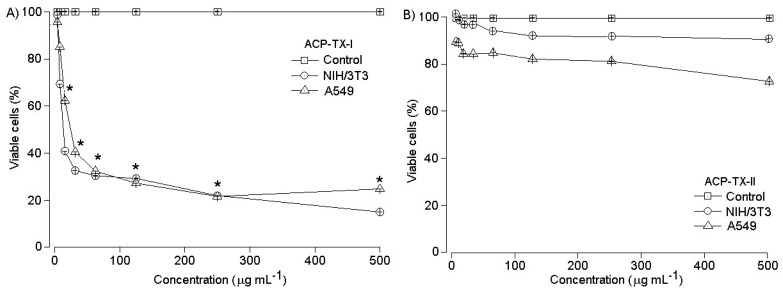
In vitro cytotoxic activity of ACP-TX-I PLA_2_ (**A**) and ACP-TX-II PLA_2_ (**B**) in NIH/3T3 (non-tumor fibroblasts) and A549 (human lung carcinoma) cells. Cell viability (%) was estimated by neutral red uptake assay. Experiments were performed in triplicate (* *p* < 0.05).

ACP-TX-I was cytotoxic to cultured NIH/3T3 (non-tumor fibroblasts) and A549 (human lung carcinoma) cells treated with different concentrations of ACP-TX-I and ACP-TX-II PLA_2_ (5–500 μg/mL) during a period of 24 h (Figure 7). ACP-TX-I PLA_2_ cytotoxicity was dose-dependent on both cell types, causing a 50% decrease in cell viability at doses ≥20 µg/mL (Figure 7A). ACP-TX-II PLA_2_ did not show toxicity in non-tumor cells used in this study, and 250–500 μg were required to decrease A549 tumor cell viability by 20 to 25% compared to controls, but the difference was not statistically significant (Figure 7B).

## 3. Discussion

Crude venom of the Trans-Pecos copperhead, *A. c. pictigaster*, was fractionated by HPLC. Fractions of interest were analyzed by MS and screened for diverse bioactivities. This work reports isolation and characterization of two basic PLA_2_s, ACP-TX-I K49 and ACP-TX-II D49, the main components of this venom (Figure 1). Using C_18_ reversed phase HPLC, 29 peaks were obtained. The two most prominent peaks, 16 and 18, named ACP-TX-I and ACP-TX-II, respectively, were selected because of PLA_2_ activity and/or molecular mass in SDS-PAGE (Figure 1 insert). Multiple PLA_2_ isoforms in the same venom were derived from accelerated microevolution, in which high mutation rates in gene coding regions, mainly associated with amino acids exposed to the solvent, allowed development of new functions [6,14].

Both ACP-TX-I and ACP-TX-II were obtained in high purity. Re-chromatography using the same chromatography system showed only one peak for each fraction (Figure S1). On SDS-PAGE, a single band was seen with an Mr of approximately 14 kDa under reducing and non-reducing conditions (Figure 1 insert). Using this purification method, several other PLA_2_ (Bp13 PLA_2_, PhTX-I, -II,-III, Bleu-PLA_2_, Bbil-TX, BbTX-II, -III, etc.) from different venoms have been purified, showing that it is rapid and efficient for the purification of these proteins in one step [6,7,8,35,36,37,38].

Molecular masses determined for ACP-TX-I (12,209.7 Da) and ACP-TX-II (14,041.1 Da), obtained with ESI mass spectrometry (Figure 2), are very close to those of PLA_2_s isolated from other snake venoms [26,34]. Partial amino acid sequencing of ACP-TX-I suggested that this protein probably has a Lys residue at position 49 (Figure 3A), based upon its high similarity to well characterized K49 PLA_2_s such as ACL, MjTX-II, BnSP-7, bl-K, and BbTX-II from *A. c. latincictus*, *B. moojeni*, *B. neuwiedi pauloensis*, *B. leucurus*, and *B. brazili*, [6,26,27,28,29]. In addition, ACP-TX-I showed negligible catalytic activity compared to crude venom and ACP-TX-II (Figure 4A).

In contrast to various homologous K49 PLA_2_s, ACP-TX-I migrated as a monomer in SDS-PAGE when analyzed under reducing and non-reducing conditions (Figure 1 insert). Recently, it was demonstrated that SDS induces oligomerization of the Lys49 PLA_2_, BPII, from *Protobothrops flavoviridis* [39], although under the same conditions, ACP-TX-I behaves as a monomer. K49 PLA_2_s isolated from three cottonmouths (*A. p. piscivorus*, *A. p. leucostoma*, and *A. p. conanti*) also behaved as monomers [14].

Partial amino acid sequencing of ACP-TX-II PLA_2_ showed that it belongs to the D49 PLA_2_ family, with an Asp residue at position 49 (Figure 3B). The catalytic site formed by H48, D49, Y52, and D89 is conserved, as reflected by the high enzymatic activity of the toxin (Figure 4A). Comparison of the amino acid sequence of ACP-TX-II shows a high degree of homology with other myotoxic D49 PLA_2_ from viperid venoms (Figure 3B). It was not possible to determine the amino acid sequence corresponding to the Ca^2+^-binding loop, comprising of residues Y24 to G35, and containing glycine residues at positions 26, 30, 32, and 33, and cysteine residues at positions 27 and 29 [40]. This domain is important to maintain Ca^2+^ in the correct position for a nucleophilic attack on the substrate.

Optimum enzymatic activity of ACP-TX-II PLA_2_ occurred at pH 8 and 37 °C (Figure 4B,D), and Ca^2+^ was an obligatory co-factor [38,41]. Ca^2+^ replacement with other divalent ions (Mg^2+^, Mn^2+^, Cd^2+^, and Zn^2+^) resulted in a loss of catalytic activity (Figure 4E) [42]. Since ACP-TX-II requires Ca^2+^ for activity, chelators such as EDTA inhibited the enzymatic activity (Figure 4F). Histidine residue alkylation also abolished enzymatic activity of ACP-TX-II PLA_2_ (Figure 4F), although alkylation does not completely disrupt the 3D structure of PLA_2_ enzymes and their capacity to bind phospholipids, but it could modify their ability to interact with specific ligands or proteins [43].

Crotapotin is the acidic moiety of crotoxin that specifically chaperones the basic PLA_2_ subunit, inhibiting its catalytic activity [44]. F5 and F6 crotapotins from *C. d. collilineatus* inhibited the enzymatic activity of ACP-TX-II PLA_2_ by approximately 55% (Figure 4F). These results are consistent with PhTX-II PLA_2_ from *Porthidium hyoprora*, which was inhibited ~60% by F2 and F3 crotapotins from *C. d. collilineatus* [8]. These results suggest that crotapotins may bind to *Agkistrodon* PLA_2_, much like how they bind to crotaline PLA_2_s.

Although snake venom PLA_2_s exhibit diverse pharmacological activities [2], myotoxicity is one of the most common effects [45]. Necrosis induced in vivo in skeletal musculature by intramuscular injection or ex vivo by incubation with differentiated skeletal muscle cells [46] is evidenced by increased plasma CK levels. ACP-TX-II PLA_2_ increased serum CK levels when injected intramuscularly (Figure 5B). Local myotoxicity is a characteristic of viperid envenomations, in which PLA_2_s affect predominantly muscles near the injection site [47]. This is consistent with clinical examinations of *Agkistrodon* bites in the United States, where the main clinical manifestations are local effects that are in some cases associated with permanent dysfunction, while systemic effects are generally absent [19].

ACP-TX-II PLA_2_ showed no systemic myotoxicity when injected intravenously. CK levels were similar to those of controls (Figure S2). Perhaps it lacks specificity and attaches to tissues at the site of injection, consistent with the hypothesis that myotoxins may act locally or systemically, proposed by Gutierrez and Ownby [46] in order to explain the pharmacological specificity of venom PLA_2_s. Myotoxic PLA_2_s bind predominantly to different cell types, as well as to muscle fibers, and are quickly sequestered after injection. On the other hand, systemic myotoxic PLA_2_s, such as PLA_2_s F6 and F6a from *Crotalus durissus collilineatus* [47], have high selectivity for skeletal muscle fibers and do not attach to other cells. This specificity allows systemic myotoxins to spread beyond the injection site, reaching the bloodstream and distant muscle cells and causing rhabdomyolysis.

Homologous K49 PLA_2_s, despite lacking catalytic activity, also cause myonecrosis when injected intramuscularly in mice [48]. Basic/hydrophobic amino acid residues located in the C-terminal region are considered one of the structural determinants for K49 myotoxicity [49,50]. To exert myotoxicity, these toxins function as obligate dimers [11]. According to this model, catalytically inactive K49 dimers undergo structural rearrangement when a membrane fatty acid enters the hydrophobic channel of one of the monomers. This reorientation aligns the C-terminal regions of the two monomers in the same plane and facilitates membrane destabilization when specific hydrophobic amino acids (Leu121, Phe125) penetrate the membrane. Once the membrane becomes disorganized, cells lose ionic control, resulting in cell death [11].

Unlike most *Bothrops* K49 PLA_2_s, ACP-TX-I does not display detectable local myotoxicity in mice (Figure 5A). Its lack of myotoxicity may reflect its monomeric structure (Figure 1 insert), preventing it from adopting different oligomeric configurations, appropriate to the physicochemical environment. These data corroborate findings with MjTX-I, which is a monomeric K49 PLA_2_ in solution isolated from *Bothrops moojeni* venom, and which shows markedly decreased myotoxic activity [51].

ACP-TX-I and ACP-TX-II induce edema, but even at high concentrations, ACP-TX-I does not reach 30% edema (Figure 6A). The marked edema induced by ACP-TX-II is likely due to phospholipid hydrolysis. This possibility is supported by studies showing that chemical modification of D49 PLA_2_s with *p*-BPB, which nullifies catalytic activity, dramatically reduces edematogenic activity of MT-III PLA_2_ from *B. asper* as well as other D49 PLA_2_s [52,53].

In addition to in vivo myotoxic and edematogenic activities, ACP-TX-I and ACP-TX-II were assayed for cytotoxicity in vitro on NIH/3T3 human fibroblasts and A549 lung cancer cells. ACP-TX-I showed high cytotoxicity in both cell lines (Figure 7A). On the other hand, even at concentrations as high as 500 μg/mL, ACP-TX-II had no inhibitory effect on NIH/3T3 fibroblasts. However, the A549 lung cancer cell line was sensitive to this myotoxin, losing ~30% viability after 24 h of incubation (Figure 7B).

The exact molecular mechanism by which snake venom PLA_2_s decrease cell viability is unknown. Some authors have proposed that cytotoxic activity on tumor cells is associated with apoptosis induction [54], and propose that PLA_2_ activity accelerates the rate of phospholipid renewal, which induces membrane changes that occur during apoptosis [55]. However, other mechanisms have been proposed: CC-PLA_2_-1 and CC-PLA_2_-2 from *Cerastes cerastes* inhibit cancerous cell adhesion and migration, as well as angiogenesis [56]. Crotoxin B interferes with signaling at epidermal growth factor receptors [57]. *Bothrops* myotoxins promote fatty acid-dependent lysis by interacting with a receptor able to activate intracellular lipase [58], etc. A K49 PLA_2_ from *Protobothrops flavoviridis* induces cell death by caspase-independent apoptosis, accompanied by rapid plasma membrane disruption in human leukemia cells. Some homologous PLA_2_s, such as MTX-II, exert cytotoxicity regardless of catalytic activity [59]. It is noteworthy that the cytotoxic mechanism depends on activities exerted by molecular regions other than the catalytic site.

## 4. Materials and Methods 

### 4.1. Venom and Reagents

Venom was obtained from the National Natural Toxins Research Center (Texas A&M University-Kingsville). Solvents and reagents used were HPLC grade, sequence grade, or high purity, obtained from Sigma, Aldrich Chemicals, Merck and BioRad.

### 4.2. Purification of ACP-TX-I and ACP-TX-II

PLA_2_ enzymes, ACP-TX-I and ACP-TX-II, from *A. c. pictigaster* venom were isolated by RP- HPLC, following Huancahuire-Vega et al. [7]. A measure of 20 mg of whole venom was dissolved in 250 µL of 0.1% TFA (buffer A) and centrifuged at 4500 g. Supernatant was then applied to an analytical RP-HPLC µ-Bondapak C_18_ column (0.78 × 30 cm; Waters 991-PDA system Milford, MA., USA), and equilibrated with buffer A for 15 min. Protein elution employed a linear gradient (0–100%, *v*/*v*) of 66.5% acetonitrile in 0.1% TFA (buffer B) at a flow rate of 1.0 mL/min. Elution was monitored at 280 nm and PLA_2_ activity was assayed in each fraction. Active PLA_2_ fractions (ACP-TX-I and II) were collected, lyophilized, and used for subsequent biochemical/functional characterization.

### 4.3. Electrophoresis

Molecular masses of ACP-TX-I and II were determined under reducing and non-reducing conditions using Tricine SDS-PAGE in a discontinuous gel and buffer system [60]. Lysozyme, soybean trypsin inhibitor, carbonic anhydrase, ovalbumin, albumin, and phosphorylase B were used as molecular mass markers.

### 4.4. Determination of Molecular Masses of the Purified Proteins by Mass Spectrometry

A measure of 4.5 µL aliquots of ACP-TX-I and ACP-TX-II PLA_2_ was injected using a C_18_ (100 µm × 100 mm) ultra-high-performance reversed phase column (nanoAcquity UPLC, Waters) coupled with nanoelectrospray tandem mass spectrometry on a Quadrupole Time-of-flight (Q-Tof) Ultima API mass spectrometer (MicroMass/Waters Milford, MA., USA) at a flow rate of 600 nL/min. The spectrometer was operated in MS continuum mode, and data acquisition was from *m*/*z* 100–3000 at a scan rate of 1 s and an interscan delay of 0.1 s. The gradient used was 0–50% acetonitrile in 0.1% formic acid over 45 min. A number of *m/z* mass spectra were accumulated over about 300 scans, and data were converted to molecular masses using maximum-entropy-based software from Masslynx 4.1 (Waters, Milford, MA., USA, 2005). Output masses ranged from 6000–20,000 Da at a 0.1 Da/channel “resolution.” The simulated isotope pattern model was used with the spectrum blur width parameter set to 0.2 Da, and minimum intensity ratios between successive peaks were 20% (left and right). After the smoothing of deconvoluted spectra, mass centroid values were obtained using 80% of the peak top and a minimum peak width at half the height of 4 channels [8].

### 4.5. Analysis of Tryptic Digests

Prior to trypsin addition (Promega sequencing grade modified), ACP-TX-I and II PLA_2_ were reduced (DTT 5 mM for 25 min to 56 °C) and alkylated (iodoacetamide, 14 mM for 30 min). After trypsin addition (20 ng/µL in Ambic 0.05 M), samples were incubated 16 h at 37 °C. The reaction was stopped with 0.4% formic acid and samples were centrifuged at 2500 rpm for 10 min. Pellets were discarded and supernatants were dried in a Speed Vac. Peptides were separated by C_18_ reverse phase chromatography (100 µm × 100 mm) (nanoAcquity UPLC, Waters, Milford, MA., USA) coupled with nanoelectrospray tandem mass spectrometry on a Q-Tof Ultima API mass spectrometer (MicroMass/Waters) at a flow rate of 600 nL/min. In order to select ions of interest, an ESI/MS mass spectrum (TOF MS mode) was acquired for each HPLC fraction before performing tandem mass spectrometry, over the mass range of 100–2000 *m*/*z*. Then, these ions were fragmented in the collision cell (TOF MS/MS mode). Raw data files from LC-MS/MS runs were processed using Masslynx 4.1 (Waters) and analyzed using the MASCOT search engine, version 2.3 (Matrix Science Ltd. London, UK) against the snake database, using the following parameters: trypsin as the enzyme, fragment mass tolerance of ±0.1 Da, peptide mass tolerance of ±0.1 Da, and oxidation as a variable modification for methionine. Sequence alignments of ACP-TX-I and ACP-TX-II with K49 and D49 PLA_2_s, respectively, were made using ClustalW in Edit Seq 5.01 © DNASTAR. (Madison, WI., USA, 2001).

### 4.6. PLA_2_ Activity

PLA_2_ activity was assayed as described by Holzer and Mackessy [61], and adapted for 96-well plates. The final volume of the standard assay mixture was 260 µL and contained 20 µL of substrate 4-nitro-3-(octanoyloxy) benzoic acid (3 mM), 200 µL of buffer (10 mM Tris–HCl, 10 mM CaCl_2_, and 100 mM NaCl, pH 8.0), 20 µL of water, and 20 µL of ACP-TX-I or ACP-TX-II (1 mg/mL). The mixture was incubated at 37 °C for 40 min, measuring absorbances at intervals of 10 min. The initial velocity (Vo) was calculated based on the value of absorbance after 20 min of reaction. ACP-TX-II was chosen by studying kinetic parameters. Different substrate concentrations (40, 20, 10, 5, 2.5, 1.0, 0.5, 0.3, 0.2, and 0.1 mM) incubated in in Tris–HCl buffer, pH 8.0 at 37 °C, were used. The optimal temperature was determined by incubating the enzyme at different temperatures. Similarly, buffers of different pHs (4–10) were used in order to determine the optimal pH. All assays were done in triplicate and absorbances at 425 nm were measured with a VersaMax 190 multiwell plate reader (Molecular Devices, Sunnyvale, CA, USA).

### 4.7. Inhibition and Chemical Modifications

The effects of EDTA and low molecular weight heparin (Mr 6.000 Da) on enzymatic activity of ACP-TX-II PLA_2_ were performed by incubating the enzyme with a 1 mM solution of EDTA or a molar ratio of 2:1 (heparin:toxin) at 37 °C for 30 min. Similarly, the effect of crotapotins F5 and F6 (1 mg/mL) from *Crotalus durissus collilineatus* upon enzymatic activity of ACP-TX-II was evaluated under the same conditions. Additionally, modification of His residues of ACP-TX-II with *p*-bromophenacyl bromide (BPB) (1.5 mg/mL in ethanol) was performed [43].

### 4.8. Myotoxic Activity

Different amounts (20 and 50 µg) of ACP-TX-I and II PLA_2_ dissolved in 100 µL of PBS were injected IM or IV, in groups of four Swiss mice (18–20 g). The control group received 100 µL of PBS. Blood was collected from the tail into the heparinized capillary tubes at different intervals (1, 3, 6, 9, and 24 h), and plasma creatine kinase (CK; EC 2.7.3.2) activity was determined by a kinetic assay (Sigma 47-UV). Activity was expressed in U/L, where one unit is defined as the phosphorylation of 1 mmol of creatine/min at 25 °C.

### 4.9. Edema-Forming Activity

Fifty µL of phosphate-buffered saline (PBS; 0.12 M NaCl, 0.04 M sodium phosphate, pH 7.2) with ACP-TX-I and II PLA_2_ (10, 20, and 50 µg/paw) were injected into the subplantar region of the right footpad of five Swiss mice (18–20 g). The left footpad received 50 µL of PBS, as a control. Immediately before inoculation (basal) and at different time intervals (1, 3, 6, 9, and 24 h) paw volume was evaluated by plethysmography (Model 7140 Plethysmometer Ugo Basile, VA., Italy). Edema-forming activity was expressed as the percentage increase in volume of the right foot pad in comparison to the left foot pad (control). The percentage of edema in toxin-inoculated paws was calculated with the equation: % edema = [((Tx × 100)/T_0_) − 100]. T_0_ is the paw volume before toxin injection. Tx is the edema (volume) measured at each time interval. The percentage of edema calculated was subtracted from the matched values at each time point in the saline injected hind paw (control) [62].

### 4.10. Cytotoxic Activity

Cytotoxic activity was assayed on NIH 3T3 fibroblasts (ATCC^®^CCL-1658™) and A549 lung cancer (ATCC^®^CCL-185™) cells, grown in plastic flasks (25 cm^2^) with RPMI 1640 medium (Cultilab, Campinas, SP, Brazil), was supplemented with 2% L-glutamine, 120 μg/mL garamycin, and 13% inactivated fetal bovine serum (complete medium). Cultures were incubated at 37 °C in an atmosphere containing 5% CO_2_. The medium was changed every 48 h, and when the culture reached confluence, subculturing was performed by treatment with trypsin and versene (Adolfo Lutz, São Paulo, SP, Brazil). Variable amounts of both ACP-TX-I and II were diluted in the assay medium and added to cells in 96-well plates. Experiments were carried out in triplicate. Cellular viability was assayed by the neutral red uptake assay of Ates et al. [63]. After treatment with toxins, the medium was removed and the culture was washed with PBS. For each well, 0.2 mL RPMI medium containing 50 µg/mL Neutral Red dye was added. The plate was incubated for 3 h at 37 °C to capture dye by viable cell lysosomes. After incubation, the medium containing the dye was removed and the wells rapidly washed with formalin-calcium to remove unincorporated dye from the cells. Then 0.1 mL of a solution of 1% (*v*/*v*) acetic acid: 50% (*v*/*v*) ethanol was added to each well to extract the dye. After shaking for 10 min on a microtitre plate shaker, absorbance was read at 540 nm. Cell viability was expressed as percentages compared to control and untreated cells.

### 4.11. Statistical Analyses

Results were reported as mean ± SEM. Dunnett’s test was used to determine the significance of differences among means by analysis of variance when several experimental groups were compared with the control group. Differences were considered statistically significant if *p* < 0.05.

## Figures and Tables

**Table 1:** Tryptic peptides of ACP-TX-I and ACP-TX-II PLA_2_. Peptides were separated and sequenced by mass spectrometry. Molecular masses are monoisotopic.

Peptides	Mass (Da) Expected	Amino Acid Sequence	Mass (Da) Calculated
**ACP-TX-I**			
1	9.864.857	GQ/KPK/QDATDR	9.864.781
2	14.075.661	DATDRCCFVHQ/K	14.076.024
3	7.753.587	VTGCDPK	7.753.535
4	14.737.322	AI/LI/LCEEK/QNPCL/IQ/K	14.737.319
5	17.537.551	MCECDK/QAVAI/LCL/IRE	17.537.619
6	11.235.574	ENL/IDTYNQ/KQ/K	11.235.509
7	9.844.505	TYWK/QYPQ/K	9.845.069
**ACP-TX-II**			
1	20.627.936	DATDRCCFVHDCCYGQ/K	20.627.754
2	15.045.434	CCFVHDCCYGQ/K	15.045.356
3	22.618.772	CCFVHDCCYGK/QI/LTACSPQ/K	22.619.149
4	17.687.631	Q/KI/LCECDRAAAI/LCFR	17.687.807
5	10.494.556	DNI/L/I/LTYDSQ/K	10.494.666
6	9.845.087	TYWKYPQ/K	9.845.069

## Supplementary Materials

The following are available online at https://www.mdpi.com/2072-6651/11/11/661/s1, Figure S1: Re-chromatography on an analytical RP-HPLC C18 analytical column of ACP-TX-I and ACP-TX-II. Figure S2: ACP-TX-II produces local myotoxicity when injected intramuscularly, but little systemic myotoxicity when injected intravenously, whereas ACP-TX-I and crude venom injected intravenously produce no systemic myotoxicity.

## References

[1] Kini R.M. (2003). Excitement ahead: Structure, function and mechanism of snake venom phospholipase A2 enzymes. Toxicon.

[2] Gutiérrez J.M., Lomonte B. (2013). Phospholipases A2: Unveiling the secrets of a functionally versatile group of snake venom toxins. Toxicon.

[3] Schaloske R.H., Dennis E.A. (2006). The phospholipase A2 superfamily and its group numbering system. Biochim. Biophys. Acta-Mol. Cell Biol. Lipids.

[4] Six D.A., Dennis E.A. (2000). The expanding superfamily of phospholipase A2 enzymes: Classification and characterization. Biochim. Biophys. Acta-Mol. Cell Biol. Lipids.

[5] Oliveira A., Bleicher L., Schrago C.G., Silva Junior F.P. (2018). Conservation analysis and decomposition of residue correlation networks in the phospholipase A2 superfamily (PLA2s): Insights into the structure-function relationships of snake venom toxins. Toxicon.

[6] Huancahuire-Vega S., Ponce-Soto L.A., Martins-de-Souza D., Marangoni S. (2009). Structural and functional characterization of brazilitoxins II and III (BbTX-II and -III), two myotoxins from the venom of Bothrops brazili snake. Toxicon.

[7] Huancahuire-Vega S., Ponce-Soto L.A., Martins-De-Souza D., Marangoni S. (2011). Biochemical and pharmacological characterization of PhTX-I a new myotoxic phospholipase A 2 isolated from Porthidium hyoprora snake venom. Comp. Biochem. Physiol. Toxicol. Pharmacol. CBP.

[8] Huancahuire-Vega S., Ponce-Soto L.A., Marangoni S. (2014). PhTX-II a basic myotoxic phospholipase A_2_ from Porthidium hyoprora snake venom, pharmacological characterization and amino acid sequence by mass spectrometry. Toxins.

[9] de Roodt A., Fernández J., Solano D., Lomonte B. (2018). A myotoxic Lys49 phospholipase A2-homologue is the major component of the venom of Bothrops cotiara from Misiones, Argentina. Toxicon.

[10] Grabner A.N., Alfonso J., Kayano A.M., Moreira-Dill L.S., Dos Santos A.P.A., Caldeira C.A.S., Sobrinho J.C., Gome A., Grabner F.P., Cardoso F.F. (2017). BmajPLA2-II, a basic Lys49-phospholipase A2 homologue from Bothrops marajoensis snake venom with parasiticidal potential. Int. J. Biol. Macromol..

[11] Fernandes C.A.H., Comparetti E.J., Borges R.J., Huancahuire-Vega S., Ponce L.A., Marangoni S., Soares A.M., Fontes M.R.M. (2013). Structural bases for a complete myotoxic mechanism: Crystal structures of two non-catalytic phospholipases A_2_-like from Bothrops brazili venom. Biochim. Biophys. Acta-Proteins Proteom..

[12] de Lima L.F.G., Borges R.J., Viviescas M.A., Fernandes C.A.H., Fontes M.R.M. (2017). Structural studies with BnSP-7 reveal an atypical oligomeric conformation compared to phospholipases A_2_-like toxins. Biochimie.

[13] Salvador G.H.M., dos Santos J.I., Lomonte B., Fontes M.R.M. (2017). Crystal structure of a phospholipase A_2_ from Bothrops asper venom: Insights into a new putative myotoxic cluster. Biochimie.

[14] Jia Y., Villarreal J. (2018). Phospholipases A_2_ purified from cottonmouth snake venoms display no antibacterial effect against four representative bacterial species. Toxicon.

[15] Angulo Y., Lomonte B. (2009). Biochemistry and toxicology of toxins purified from the venom of the snake Bothrops asper. Toxicon.

[16] Campbell J. (2004). The Venomous Reptiles of the Western Hemisphere.

[17] Guiher T.J., Burbrink F.T. (2008). Demographic and phylogeographic histories of two venomous North American snakes of the genus Agkistrodon. Mol. Phylogenet. Evol..

[18] Knight R.A. (1991). Molecular Systematics of the Agkistrodon Complex.

[19] Domanski K., Kleinschmidt K.C., Greene S., Ruha A.M., Berbata V., Onisko N., Campleman S., Brent J., Wax P. (2019). Cottonmouth snake bites reported to the ToxIC North American snakebite registry 2013–2017. Clin. Toxicol..

[20] Walter F.G., Stolz U., Shirazi F., Walter C.M., McNally J. (2012). Epidemiology of the reported severity of copperhead (Agkistrodon contortrix) snakebite. South. Med. J..

[21] Johnson E.K., Ownby C.L. (1993). Isolation of a myotoxin from the venom of Agkistrodon contortrix laticinctus (broad-banded copperhead) and pathogenesis of myonecrosis induced by it in mice. Toxicon.

[22] Bocian A., Urbanik M., Hus K., Łyskowski A., Petriaal V., Andrejčáková Z., Petrillová M., Legáth J. (2016). Proteomic analyses of Agkistrodon contortrix contortrix venom using 2D electrophoresis and MS techniques. Toxins.

[23] Fořtová H., Suttnar J., Dyr J.E., Pristach J. (1997). Simultaneous isolation of protein C activator, fibrin clot promoting enzyme (fiprozyme) and phospholipase A_2_ from the venom of the southern copperhead snake. J. Chromatogr. B Biomed. Appl..

[24] Lucena S., Rodríguez-Acosta A., Grilli E., Alfonso A., Goins A., Ogbata I., Walls R., Suntravat M., Uzcátegui N.L., Guerrero B. (2016). The characterization of trans-pecos copperhead (Agkistrodon contortrix pictigaster) venom and isolation of two new dimeric disintegrins. Biologicals.

[25] Lomontea B., Tsai W.-C., Ureña-Diaz J.M., Sanz L., Mora-Obando D., Sánchez E.E., Fry B.G., Gutiérreza J.M., Gibbs H.L., Sovic G.M. (2014). Venomics of New World pit vipers: Genus-wide comparisons of venom proteomes across Agkistrodon. J. Proteom..

[26] De Araujo H.S.S., White S.P., Ownby C.L. (1996). cDNA cloning and sequence analysis of a lysine-49 phospholipase A 2 myotoxin from Agkistrodon contortrix laticinctus snake venom. Arch. Biochem. Biophys..

[27] Soares A.M., Rodrigues V.M., Homsi-Brandeburgo M.I., Toyama M.H., Lombardi F.R., Armo R.K., Giglio J.R. (1998). A rapid procedure for the isolation of the LYS-49 myotoxin II from bothrops moojeni (caissaca) venom: Biochemical characterization, crystallization, myotoxic and edematogenic activity. Toxicon.

[28] Soares A.M., Guerra-Sá R., Borja-Oliveira C.R., Rodrigues V.M., Rodrigues-Simioni L., Rodrigues V., Fontes M.R.M., Lomonte B., Gutiérrez J.M., Giglio J.R. (2000). Structural and functional characterization of BnSP-7, a Lys49 myotoxic phospholipase A_2_ homologue from Bothrops neuwiedi pauloensis venom. Arch. Biochem. Biophys..

[29] Higuchi D.A., Barbosa C.M.V., Bincoletto C., Chagas J.R., Magalhaes A., Richardson M., Sanchez E.F., Pesquero J.B., Araujo R.C., Pesquero J.L. (2007). Purification and partial characterization of two phospholipases A2 from Bothrops leucurus (white-tailed-jararaca) snake venom. Biochimie.

[30] Lathrop B.K., Burack W.R., Biltonen R.L., Rule G.S. (1992). Expression of a group II phospholipase A2 from the venom of Agkistrodon piscivorus piscivorus in Escherichia coli: Recovery and renaturation from bacterial inclusion bodies. Protein Expr. Purif..

[31] Welches W., Reardon I., Heinrikson R.L. (1993). An examination of structural interactions presumed to be of importance in the stabilization of phospholipase A_2_ dimers based upon comparative protein sequence analysis of a monomeric and dimeric enzyme from the venom of Agkistrodon p. piscivorus. J. Protein Chem..

[32] Chijiwa T., Tokunaga E., Ikeda R., Terada K., Ogawa T., Oda-Ueda N., Hattori S., Nozaki M., Ohno M. (2006). Discovery of novel [Arg49] phospholipase A_2_ isozymes from *Protobothrops elegans* venom and regional evolution of Crotalinae snake venom phospholipase A_2_ isozymes in the southwestern islands of Japan and Taiwan. Toxicon.

[33] Wang Y., Cui G., Zhao M., Yang J., Wang C., Giese R., Peng S. (2008). Bioassay-directed purification of an acidic phospholipase A 2 from Agkistrodon halys pallas venom. Toxicon.

[34] Pan H., Liu X., Ou-Yang L., Yang G., Zhou Y., Li Z., Wu X. (1998). Diversity of cDNAs encoding phospholipase A 2 from Agkistrodon halys Pallas venom, and its expression in E. coli. Toxicon.

[35] Marques P.P., Esteves A., Lancellotti M., Ponce-Soto L.A., Marangoni S. (2015). Novel acidic phospholipase A_2_ from Porthidium hyoprora causes inflammation with mast cell rich infiltrate. Biochem. Biophys..

[36] Marangoni F.A., Ponce-Soto L.A., Marangoni S., Landucci E.C.T. (2013). Unmasking snake venom of bothrops leucurus: Purification and pharmacological and structural characterization of new PL A2 Bleu TX-III. Biomed. Res. Int..

[37] Victor C.C., Floriano R.S., Rodrigues-Simioni L., Winck F.V., Baldasso P.A., Ponce-Soto L.A., Marangoni S. (2013). Biochemical, pharmacological, and structural characterization of new basic PLA2 Bbil-TX from bothriopsis bilineata snake venom. Biomed. Res. Int..

[38] Sucasaca-Monzón G., Randazzo-Moura P., Rocha T., Torres-Huaco F.D., Vilca-Quispe A., Alberto Ponce-Soto L., Marangoni S., da Cruz-Höfling M.A., Rodrigues-Simioni L. (2015). Bp-13 PLA2: Purification and neuromuscular activity of a new Asp49 toxin isolated from bothrops pauloensis snake venom. Biochem. Res. Int..

[39] Matsui T., Kamata S., Ishii K., Maruno T., Ghanem N., Uchiyama S., Kato K., Suzuki A., Oda-Ueda N., Ogawa T. (2019). SDS-induced oligomerization of Lys49-phospholipase A_2_ from snake venom. Sci. Rep..

[40] Singh G., Gourinath S., Sharma S., Paramasivam M., Srinivasan A., Singh T.P. (2001). Sequence and crystal structure determination of a basic phospholipase A2 from common krait (Bungarus caeruleus) at 2.4 A resolution: Identification and characterization of its pharmacological sites. J. Mol. Biol..

[41] Diz Filho E.B.S., Marangoni S., Toyama D.O., Fagundes F.H.R., Oliveira S.C.B., Fonseca F.V., Calgarotto A.K., Joazeiro P.P., Toyama M.H. (2009). Enzymatic and structural characterization of new PLA2 isoform isolated from white venom of Crotalus durissus ruruima. Toxicon.

[42] Yu B.Z., Jain M.K., Berg O.G. (1993). The Divalent Cation Is Obligatory for the Binding of Ligands to the Catalytic Site of Secreted Phospholipase A_2_. Biochemistry.

[43] Huancahuire-Vega S., Correa D.H.A., Hollanda L.M., Lancellotti M., Ramos C.H.I., Ponce-Soto L.A. (2013). Chemical modifications of PhTX-I myotoxin from porthidium hyoprora snake venom: Effects on structural, enzymatic, and pharmacological properties. Biomed. Res. Int.

[44] Aird S.D., Kaiser I.I., Lewis R.V., Kruggel W.G. (1985). Rattlesnake presynaptic neurotoxins: Primary structure and evolutionary origin of the acidic subunit. Biochemistry.

[45] Montecucco C., Gutiérrez J.M., Lomonte B. (2008). Cellular pathology induced by snake venom phospholipase A2 myotoxins and neurotoxins: Common aspects of their mechanisms of action. Cell. Mol. Life Sci..

[46] Gutiérrez J.M., Ownby C.L. (2003). Skeletal muscle degeneration induced by venom phospholipases A_2_: Insights into the mechanisms of local and systemic myotoxicity. Toxicon.

[47] Gutiérrez J.M., Alberto Ponce-Soto L., Marangoni S., Lomonte B. (2008). Systemic and local myotoxicity induced by snake venom group II phospholipases A_2_: Comparison between crotoxin, crotoxin B and a Lys49 PLA2 homologue. Toxicon.

[48] Gutiérrez J.M., Lomonte B., Chaves F., Moreno E., Cerdas L. (1986). Activities of a Toxic a Isolated From the Venom of the Snake Bothrops. Comp. Biochem. Physiol. Part C: Comp. Pharmacol..

[49] Lomonte B., Angulo Y., Calderón L. (2003). An overview of lysine-49 phospholipase A2 myotoxins from crotalid snake venoms and their structural determinants of myotoxic action. Toxicon.

[50] Lomonte B., Rangel J. (2012). Snake venom Lys49 myotoxins: From phospholipases A_2_ to non-enzymatic membrane disruptors. Toxicon.

[51] Salvador G.H.M., Fernandes G.A.H., Magro A.J., Marchi-Salvador D.P., Cavalcante W.L.G., Fernandez R.M., Gallacci M., Soares A.M., Oliveira C.L.P., Fontes M.R.M. (2013). Structural and Phylogenetic Studies with MjTX-I Reveal a Multi-Oligomeric Toxin—a Novel Feature in Lys49-PLA2s Protein Class. PLoS ONE.

[52] Chaves F., León G., Alvarado V.H., Gutiérrez J.M. (1998). Pharmacological modulation of edema induced by Lys-49 and Asp-49 myotoxic phospholipases A_2_ isolated from the venom of the snake Bothrops asper (terciopelo). Toxicon.

[53] Zuliani J.P., Fernandes C.M., Zamuner S.R., Gutiérrez J.M., Teixeira C.F.P. (2005). Inflammatory events induced by Lys-49 and Asp-49 phospholipases A_2_ isolated from Bothrops asper snake venom: Role of catalytic activity. Toxicon.

[54] Chen K.C., Kao P.H., Lin S.R., Sen Chang L. (2008). p38 MAPK activation and mitochondrial depolarization mediate the cytotoxicity of Taiwan cobra phospholipase A_2_ on human neuroblastoma SK-N.-SH cells. Toxicol. Lett..

[55] Panini S.R., Yang L., Rusinol A.E., Sinensky M.S., Bonventre J.V., Leslie C.C. (2001). Arachidonate metabolism and the signaling pathway of induction of apoptosis by oxidized LDL/oxysterol. J. Lipid Res..

[56] Zouari-Kessentini R., Luis J., Karray A., Kallech-Ziri O., Srairi N., Bazaa A., Loret E., Sofiane B., Mohamed E., Marrakchi N. (2009). Two purified and characterized phospholipases A2 from Cerastes cerastes venom, that inhibit cancerous cell adhesion and migration. Toxicon.

[57] Donato N.J., Martin C.A., Perez M., Newman R.A., Vidal J.C., Etcheverry M. (1996). Regulation of epidermal growth factor receptor activity by crotoxin, a snake venom phospholipase A_2_ toxin: A novel growth inhibitory mechanism. Biochem. Pharmacol..

[58] Gebrim L.C., Marcussi S., Menaldo D., de Menezes C.S.R., Nomizo A., Hamaguchi A., Silveira-Lacerda E.P., Homsi-Brandeburgo A.I., Sampaio S.V., Soares A.M. (2009). Antitumor effects of snake venom chemically modified Lys49 phospholipase A2-like BthTX-I and a synthetic peptide derived from its C-terminal region. Biologicals.

[59] Costa T.R., Menaldo D.L., Oliveira C.Z., Santos-Filho N.A., Teixeira S.S., Nomizo A., Fuly A.L., Monteiro M.C., Souza B.M., Palma M.S. (2008). Myotoxic phospholipases A2 isolated from Bothrops brazili snake venom and synthetic peptides derived from their C-terminal region: Cytotoxic effect on microorganism and tumor cells. Peptides.

[60] Schägger H., von Jagow G. (1987). Tricine-sodium dodecyl sulfate-polyacrylamide gel electrophoresis for the separation of proteins in the range from 1 to 100 kDa. Anal. Biochem..

[61] Holzer M., Mackessy S.P. (1996). An aqueous endpoint assay of snake venom phospholipase A_2_. Toxicon.

[62] Ferreira T., Camargo E.A., Ribela M.T.C.P., Damico D.C., Marangoni S., Antunes E., Nucci G.D., Landucci E.C.T. (2009). Inflammatory oedema induced by Lachesis muta muta (Surucucu) venom and LmTX-I in the rat paw and dorsal skin. Toxicon.

[63] Ates G., Rogiers T.V., Rodrigues R.M. (2017). Assaying Cellular Viability Using the Neutral Red Uptake Assay. Cell Viability Assays.

