# Supplementary material for: ACP-TX-I and ACP-TX-II, Two Novel Phospholipases A2 Isolated from Trans-Pecos Copperhead Agkistrodon contortrix pictigaster Venom: Biochemical and Functional Characterization
Source: Toxins (Basel). 2019 Nov 14;11(11):661. doi: 10.3390/toxins11110661 (PMC6891687; doi:10.3390/toxins11110661)
Supplement: Supplementary file 1 [file toxins-11-00661-s001.pdf]

Supplementary Materials: ACP-TX-I and ACP-TX-II, Two Novel Phospholipases A2 Isolated from Trans-Pecos Copperhead *Agkistrodon contortrix pictigaster* Venom: Biochemical and Functional Characterization

Salomón Huancahuire-Vega, Luciana M. Hollanda, Mauricio Gomes-Heleno, Edda E. Newball-Noriega and Sergio Marangoni

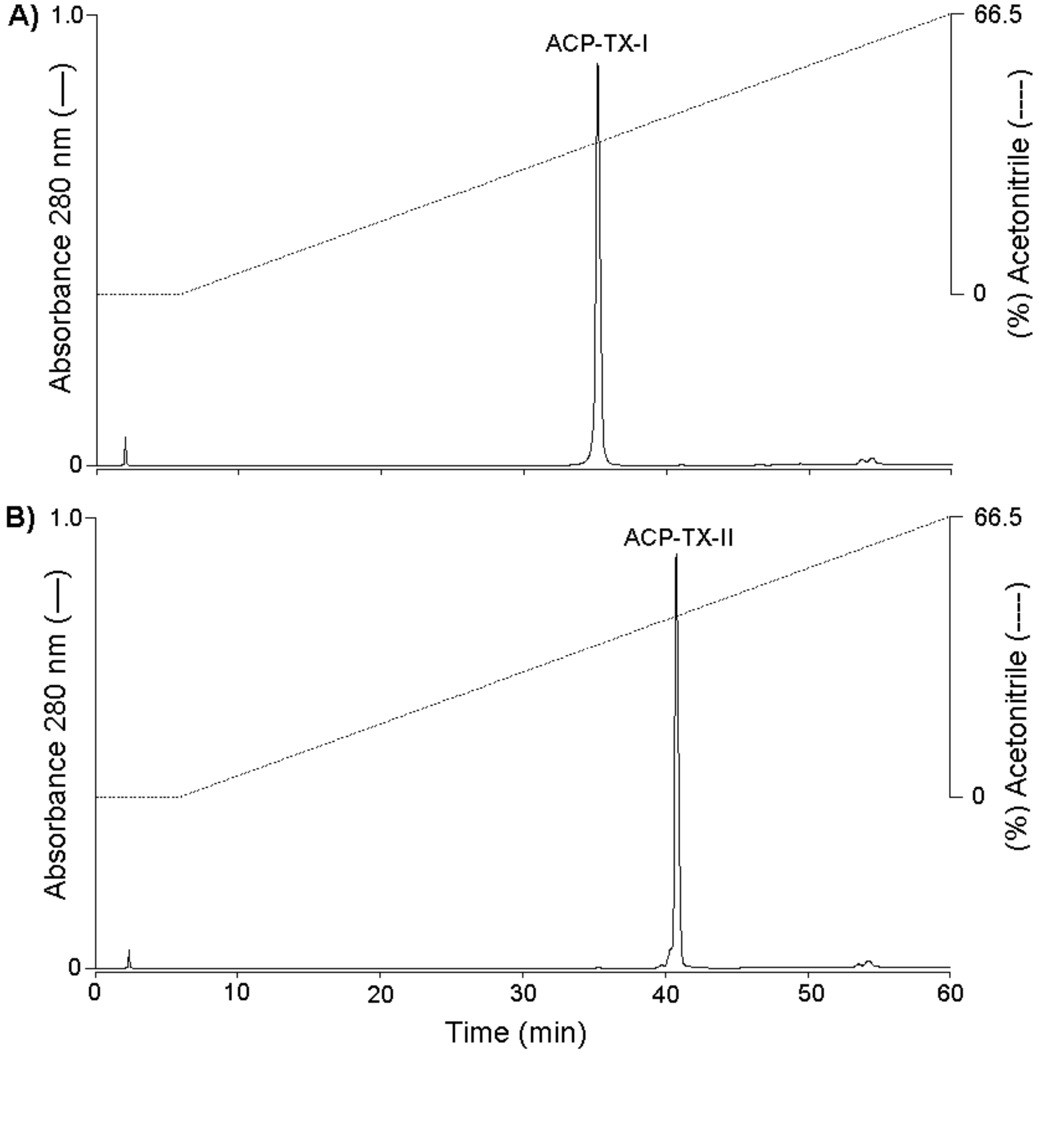

Figure S1. Re-chromatography on an analytical RP-HPLC C_{18} analytical column of ACP-TX-I (**A**) and ACP-TX-II (**B**). Protein elution employed a linear gradient (0–66.5%) of acetonitrile at a flow rate of 1.0 mL/min. Elution was monitored at 280 nm.

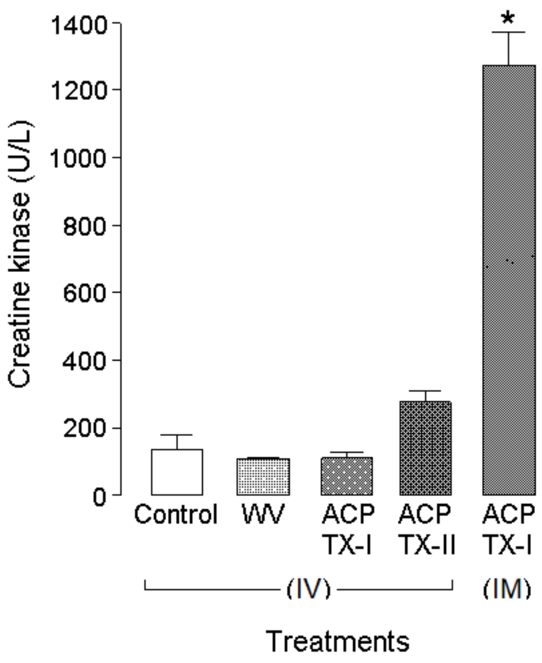

Figure S2. ACP-TX-II produces local myotoxicity when injected intramuscularly, but little systemic myotoxicity when injected intravenously, whereas ACP-TX-I and crude venom injected intravenously produce no systemic myotoxicity. 100 μg of *A. contortrix pictigaster* venom (WV), ACP-TX-I and ACP-TX-II dissolved in 100 μL of PBS were injected intravenously (IV) in mice (tail vein). The control group received 100 μL of PBS. Blood was collected from the tail into heparinized capillary tubes 3 h after administration of venom and toxins and plasma creatine kinase activity (CK in U/L) was determined. Plasma CK levels did not increase significantly compared to control. For comparison, the last column represents the an intramuscular injection (IM) of the same concentration of ACP-TX-II, the increased plasma CK levels above 1000 U/L. Each column represent means ± SD of four mice per group. (* $p < 0.05$).
